# Shallow Learning Techniques for Early Detection and Classification of Cyberattacks over MQTT IoT Networks

**DOI:** 10.3390/s26020468

**Published:** 2026-01-10

**Authors:** Antonio Díaz-Longueira, Jose Aveleira-Mata, Álvaro Michelena, Andrés-José Piñón-Pazos, Óscar Fontenla-Romero, José Luis Calvo-Rolle

**Affiliations:** 1 Department of Industrial Engineering, University of A Coruña, Ciencia y Técnica Cibernética, Centro de Investigación de Tecnologías de la Información y de la Comunicación, 15403 Ferrol, Spain; a.diazl@udc.es (A.D.-L.); andres.pinon@udc.es (A.-J.P.-P.); jose.rolle@udc.es (J.L.C.-R.); 2 SECOMUCI Research Group, Department of Electric, Systems and Automatics Engineering, Universidad de León, 24008 León, Spain; jose.aveleira@unileon.es; 3 Laboratorio de Investigación y Desarrollo en Inteligencia Artificial, Faculty of Computer Science, University of A Coruña, Centro de Investigación de Tecnologías de la Información y de la Comunicación, 15071 A Coruña, Spain; oscar.fontenla@udc.es

**Keywords:** MQTT protocol, cybersecurity, shallow learning, DoS attack, unauthorized client attack, multiclass classification

## Abstract

**Highlights:**

**What are the main findings?**

- This work proposes a lightweight multiclassifier for cyberattack detection on MQTT networks. The classifier, based on shallow learning techniques, enables the deployment in resorce-constrained devices, targeting Denial-of-Service and Intrusion attacks in IoT MQTT environments.
- Development of an intrusion detector system based on shallow learning techniques.

**What is the implication of the main finding?**

- The detection and classification of cyberattacks on IoT networks over MQTT is enabled.

**Abstract:**

The increasing global connectivity, driven by the expansion of the Internet of Things (IoT), is generating a significant increase in system vulnerabilities. Cyberattackers exploit the computing and processing limitations of typical IoT devices and take advantage of inherent vulnerabilities in wireless networks and protocols to attack networks, compromise infrastructure, and cause damage. This paper presents a shallow learning multiclassifier approach for detecting and classifying cyberattacks on IoT networks. Specifically, it addresses MQTT networks, widely used in the IoT, to detect Denial-of-Service (DoS) and Intrusion attacks, using inter-device communication data as a basis. The use of shallow learning techniques allows this cybersecurity system to be implemented on resource-constrained devices, enabling local network monitoring and, consequently, increasing security and incident response capabilities by detecting and identifying attacks. The proposed system is validated on a real dataset obtained from an IoT system over MQTT, demonstrating its correct operation by achieving an accuracy greater than 99% and F1-score greater than 80% in the detection of Intrusion attacks.

## 1. Introduction

The Internet of Things (IoT) is rapidly transforming both everyday life and large industrial systems. By interconnecting hundreds of devices and enabling real-time data exchange, IoT solutions extend the functionality of common devices through connectivity to the Internet, sensors, and actuators. These technologies already support applications ranging from medical and health devices to Industry 4.0 systems and smart city infrastructure [1]. In these contexts, IoT platforms are integrated with control systems to enable remote monitoring and real-time operation from different client devices, making security in Industry 4.0 environments a critical issue [2].

IoT devices are characterized by significant limitations: they must be energy efficient and run on modest processors, requiring reduced computing power [3]. To address these constraints, lightweight protocols are used. At the application layer, MQTT, CoAP, XMPP, and DDS stand out [4], while technologies such as Bluetooth, BLE, 6LoWPAN, ZigBee (IEEE 802.15.4) and LoRa are used for physical or link connectivity [5]. These architectures enable low-power communications that reduce congestion and facilitate near real-time interaction. The growth of this ecosystem is remarkable: by 2034, it is estimated that there will be 40,603 millions connected devices [6]. This magnitude, coupled with the diversity of protocols and hardware limitations, intensifies security challenges. Attackers can compromise vulnerable devices and form botnets to execute distributed denial-of-service (DDoS) attacks. Notable examples include the Mirai botnet, which controlled some 400,000 IoT devices [7], and Dark Nexus, detected in 2020 with 1372 active nodes [8].

Among these lightweight protocols, MQTT stands out as the de facto standard for IoT messaging. Its simplicity, small code footprint, and efficient use of bandwidth make it particularly suitable for constrained devices operating in resource-limited environments. MQTT follows a broker-centric publish/subscribe paradigm that reduces device complexity and enables scalable communication across large numbers of clients. As a result, it has become one of the most widely deployed protocols in smart city infrastructures, industrial automation, and consumer IoT products, often preferred over alternatives such as CoAP or XMPP due to its robustness, ease of integration, and support for diverse Quality of Service (QoS) levels. However, the same characteristics that favor its adoption (lightweight design and limited security features by default) also expose specific vulnerabilities that attackers can exploit, underscoring the need for focused security studies on MQTT-based systems.

Attackers use multiple vectors to introduce malware and turn devices into bots: default configurations or insecure gateways. This type of threat has been demonstrated in different IoT areas, such as BLE [9], commercial devices [10], and popular protocols such as CoAP [11] and MQTT [12]. In critical infrastructures, such as the energy sector, DDoS attacks are particularly dangerous, as they can leave large populations without electricity [13,14]. To mitigate these risks, various studies have investigated techniques for anomaly detection [15] and defenses against false data injections [16]; measures based on active authentication [17] have also been proposed.

The actors involved in cyberattacks in general, and IoT cyberattacks in particular, exhibit considerable variation in both their nature and motivation. While not entirely separate, the perpetrators can be categorized as individuals, organized groups, and intelligence agencies, while the reasons and motivations behind these attacks can include obtaining financial gain, accessing sensitive and confidential information, or negatively impacting the operation of critical infrastructure [18].

To counter these threats, Intrusion Detection Systems (IDSs) provide a practical and non-intrusive solution, since they do not require altering the configuration of IoT devices or the underlying networks. However, rule-based IDSs (e.g., Suricata, Snort) are challenging to adapt to IoT environments, where the continuous appearance of new devices and vulnerabilities requires constant rule updates [19]. For this reason, anomaly-based IDSs have gained increasing attention: by modeling normal behavior and detecting deviations, they can identify novel attacks. Machine learning and soft computing approaches are commonly applied in this domain, as they allow IDS models to be trained with datasets containing both benign and malicious traffic [20]. In particular, shallow learning models are attractive for the IoT due to their low computational cost, enabling real-time deployment on resource-constrained devices [20,21,22]. Typical IoT devices have significant limitations in computing speed and capacity, severe memory and latency constraints, and critical power consumption levels, as they are often battery-powered. All these limitations make the design and deployment of deep learning models on these devices impractical. Using shallow learning techniques allows for the implementation of IDSs on these devices, as they offer short training and prediction times and a lightweight operating environment, and therefore require less computing power.

Several datasets have been used to train and evaluate IDSs in IoT contexts. Among the classic benchmarks are KDD and NSL-KDD [23], which, although not specific to IoT, have been used to study DoS and DDoS attacks [24,25]. For wireless networks, the AWID dataset provides detailed IEEE 802.11 traffic and has been used in Wi-Fi–based IoT studies [26,27]. IoT-oriented datasets include IoT-23, which contains DNS traffic for botnet detection [28]. Several MQTT datasets also exist, though many do not cover vulnerabilities specific to the protocol. For example, MQTT-IoT-IDS2020 captures traffic from a simulated environment with brute-force and scanning attacks [29], while MQTTSet includes brute force, malformed data, and DoS attacks in a simulated testbed [30]. Another relevant dataset is Bot-IoT, which includes DoS/DDoS attacks together with benign MQTT traffic that simulates a weather station, allowing analysis of realistic attack impacts [31,32].

MQTT remains one of the most widely adopted IoT protocols due to its lightweight publish/subscribe design, but exhibits vulnerabilities that adversaries can exploit [33], with MitM attacks being particularly difficult to detect. In previous work, the authors generated a dataset by capturing real traffic in an MQTT-based IoT environment, which included complete network activity [34]. This dataset was later used to achieve promising results with autoencoder techniques [35]. A subsequent study applied a multiclass classification approach to detect Intrusion, DoS, and MitM attacks, selecting features with an entropy-based criterion. Although the model was effective for DoS detection, its performance in Man-in-the-Middle and Intrusion attacks was more limited [36]. In other related work, in [37], the authors propose the use of deep learning methods to implement an intrusion detection system in an IoT network. This work, which detects various cyberattacks with a high degree of confidence, employs different neural network models for multiclassification. In another similar publication, [38], an optimized deep learning method is used that enables real-time detection of cyberattacks in IoT infrastructures. The detection, which is accurate for different threats, is performed by an external device connected to the network, which handles the processing and inference of the models.

This work proposes a simple, lightweight, and efficient model for the multiclassification of cyberattack on MQTT networks. The classifier, based on shallow learning techniques, enables the deployment in resource-constrained devices, targeting Denial-of-Service and Intrusion attacks in IoT MQTT environments. This IDS is designed to be run by typical IoT network devices, enabling early detection of cyberattacks at the edge. The main contribution of this work is:

- Enabling the detection and classification of cyberattacks on the MQTT network devices themselves.

To ensure the device functions correctly, even when running a machine learning model designed to detect and classify cyberattacks in edge environments, it is essential that the model exhibit lightweight behavior. This implies minimal memory and storage consumption, considering the inherent limitations of this type of device. Likewise, the model must be computationally efficient, reducing CPU usage time and optimizing resource management, which contributes to maximizing both energy savings and resource availability for other tasks, such as communication. Finally, in terms of training and inference phases, since training is performed on external equipment, this aspect does not represent a critical constraint in the IoT device environment. However, inference time is a relevant and critical parameter, so it must be as short as possible to guarantee a fast and efficient response. Since data quality is critical for this type of approach, the study relies on real MQTT traffic captures that reflect realistic network conditions [34].

The remainder of the manuscript is organized as follows. Section 2 describes the case study, detailing the testing environment and the dataset used. Section 3 presents the different techniques implemented in our proposal. Section 4 details the parameter configurations. Section 5 presents the evaluation metrics and the comparative results. Finally, Section 6 summarizes the conclusions and possible lines of future work.

## 2. Case Study

MQTT (Message Queuing Telemetry Transport) is a lightweight publish/subscribe protocol widely adopted in constrained networks and devices. Communication follows a star topology where clients connect to a central broker. Publishers send data to specific topics, and subscribers receive only the messages from the topics they follow. The broker is responsible for routing messages, maintaining sessions, and enforcing quality-of-service levels (QoS 0, 1, 2). This design decouples publishers from subscribers and reduces the complexity at the device level, making MQTT highly suitable for IoT deployments [39].

This case study describes the construction of an MQTT-centered dataset generated in a controlled LAN, capturing real traffic under normal conditions and during two distinct attack categories at the protocol layer: denial of service, and unauthorized client access. The attack scenarios chosen are consistent with the MQTT specification and practical deployments [40]. Examples include excessive connection or publish storms with oversized payloads, on-path packet manipulation, and wildcard subscriptions that expose entire topic hierarchies. By focusing strictly on the protocol layer, the dataset avoids dependencies on vendor-specific implementations and directly reflects realistic MQTT threats.

### 2.1. Environment Overview

**Figure 1:**
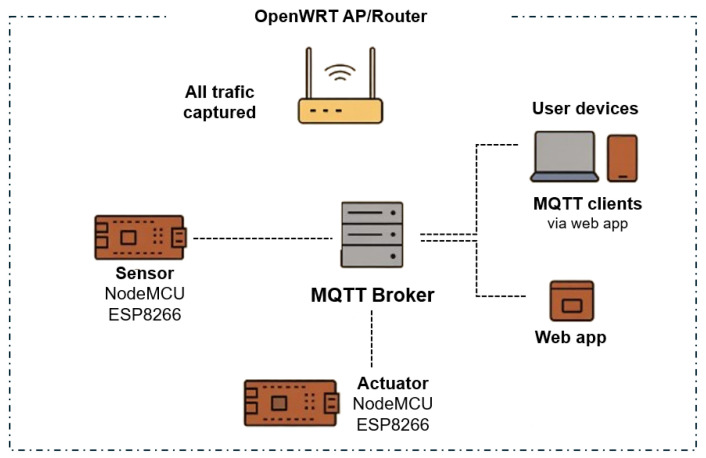
Local WLAN with two IoT nodes and different SER devices representing the MQTT environment used to obtain the dataset.

The experimental setup was deployed on a local WLAN. A TP-Link TL-WR1043ND router running OpenWRT acted both as the access point and as the traffic capture unit through tcpdump. The broker was implemented in Node.js using the Aedes library, while a companion web interface built with mqtt.js enabled monitoring and interaction. The IoT nodes consisted of NodeMCU ESP8266 boards, one equipped with an ultrasonic sensor (HC-SR04) and another with a relay-based actuator. Both devices exchanged telemetry and control data through MQTT topics such as distance/ultrasonic and light/relay, as illustrated in Figure 1. The WLAN user devices generated background traffic, ensuring co-existence with the MQTT flows. OpenWRT allowed the centralization and capture of all network traffic, which was stored in PCAP files for later processing.

### 2.2. Protocol-Level MQTT Attack Scenarios

This section details the two different attacks implemented, all of which exploit inherent properties of the publish/subscribe model and MQTT’s security framework. These scenarios focus on the broker-centered architecture, optional security mechanisms, and message handling rules, without requiring exploitation of device firmware or operating system vulnerabilities.

**Denial of Service (DoS) against the broker.** The central broker can be overwhelmed by protocol-level abuses such as excessive client connections, bursts of PUBLISH messages, persistent sessions, or abnormally large payloads. In our experiments, a client simulator generated floods of publishers at high rates, leading to resource exhaustion on the broker. Previous studies confirm that such behaviors represent protocol-level misuse rather than implementation flaws, suggesting countermeasures such as connection quotas, payload limits, and back-pressure mechanisms [41].

**Intruding client/wildcard subscription.** If the broker is misconfigured to allow unrestricted access, an adversary can connect as a legitimate client and subscribe with the wildcard character “#”, thereby receiving every message across all topics. This capability is part of MQTT’s topic filtering system, which requires explicit authorization and fine-grained policies to prevent abuse. Global scans report that thousands of MQTT services remain publicly exposed on TCP/1883, many of them without authentication [42,43]. In our environment, the attacker subscribed with “#” and successfully monitored legitimate topics (e.g., distance/ultrasonic, light/relay) while injecting unauthorized traffic. The selection of a controlled environment with homogeneous nodes isolates attack signatures within the MQTT protocol layer. Since the analyzed vulnerabilities, such as Denial of Service and unauthorized wildcard access, exploit the internal logic of the publish/subscribe model, their behavior remains independent of hardware diversity or the coexistence of other network protocols. This approach ensures that the dataset contains significant frames for model training by reducing statistical noise. Furthermore, this isolation methodology is compatible with other datasets generated under the same standard, such as CoAP [44], enabling the integration of data for future multi-protocol detection models.

### 2.3. Data Acquisition and Dataset Characteristics

Traffic was captured directly from the WLAN through the OpenWrt router, stored as PCAP files, and subsequently processed with a parsing tool that filtered and labeled the records. The final schema includes 67 attributes grouped into generic network features and MQTT-specific fields (following the Wireshark Display Filter Reference). Each entry is labeled into one of three categories: normal, DoS, or Intrusion.

The dataset is organized into two CSV files, each corresponding to one attack scenario (Table 1).

The complete dataset is publicly available on Figshare https://figshare.com/s/7421593360b8fdccb306 (accessed on 4 October 2025), accompanied by documentation and integrity checksums. All captures were performed on a controlled testbed and do not contain personal data.

## 3. Implemented Techniques

The methods and techniques used in the development of the cyberattack multiclassifier are briefly presented and described below. First, Section 3.1 presents the over-sampling technique used; and second, Section 3.2 presents the machine learning techniques implemented for the multiclass classification task.

### 3.1. Over-Sampling Techniques

Over-sampling techniques are designed to generate new samples from minority classes. In this way, by creating samples of underrepresented classes, an unbalanced classification problem becomes a balanced one, where all classes are equally represented.

#### Synthetic Minority Over-Sampling Technique

Synthetic Minority Over-sampling Technique (SMOTE) [45] is an over-sampling technique that generates new synthetic samples from minority classes. Its operating method is based on exploring the feature space, selecting samples from the underrepresented class, and creating new samples, belonging to this minority class, on the lines that join any or all of the original samples with the K closest samples belonging to that same class. That is, for each sample, the k closest samples, with the same class, are selected, and samples are generated in the joining direction. The number of samples generated will depend on the data set and its imbalance.

### 3.2. Classification Techniques

The chosen classification techniques respond to the need to implement lightweight and shallow techniques that allow rapid execution without excessive energy expenditure on devices with limited resources without neglecting performance and the ability to correctly detect and classify cyberattacks on IoT communications.

#### 3.2.1. Shallow Learning

Shallow Learning refers to a set of machine learning techniques, algorithms, and methods characterized by simple, shallow architectures limited to a few processing layers [46]. Compared to Deep Learning, currently widely used in fields such as computer vision, robotics, and generative artificial intelligence, these techniques have considerably lower energy and processing requirements, making them suitable for low-complexity problems. Various authors [46,47] consider methods proposed before 2006, the year Deep Learning became popular, to be Shallow Learning. Shallow Learning methods are notable for their high explainability and simplicity, achieving competitive performance on well-defined problems with limited datasets, short training and inference times, and low energy consumption.

Due to the low energy consumption and reduced computing capacity typical of IoT devices, the possibility of including easily executable ML models that allow the detection of cyberattacks at the Edge without altering the speed and communication capacity makes the use of these methods to create a multiclassifier capable of detecting cyberattacks in an IoT network of particular interest.

#### 3.2.2. Linear Discriminant Analysis

Linear Discriminant Analysis (LDA), or Fischer Linear Discriminant Analysis, is a statistical method widely used for supervised classification tasks. It is characterized by generating linear decision surfaces, its low computational cost, and the fact that there is no hyperparameters to tune. Its operation is based on determining the linear combination that maximizes the separation between the classes of the training data. To achieve this, the LDA technique seeks the direction *w* in the feature space that maximizes the distance between the means of each class, while minimizing the standard variance in each class. Equation (1) expresses the separability function for the direction [48].

(1) $$J\left(w\right)=\frac{w^{T}\times \mu \times w}{w^{T}\times \sigma \times w}$$

where μ is the difference in means between classes and σ is the variance within the classes.

This method works on the assumption that the classes have the same covariance matrices [49].

#### 3.2.3. QuadraticDiscriminant Analysis

Quadratic Discriminant Analysis (QDA), in contrast to Linear Discriminant Analysis, Section 3.2.2, is an algorithm that generates models of a quadratic (nonlinear) decision surface. In this technique, the assumption made by LDA that the covariance matrices between classes were equal is relaxed, assuming that these matrices are distinct [50,51].

#### 3.2.4. Naive Bayes

Naive Bayes (NB) is a family of statistical methods based on Bayes’ theorem. Furthermore, known as Naive Bayesian, these classifiers work with the "naive" assumption of conditional independence between pairs of features for a given class. Although this assumption is rarely met in practice, NB classifiers typically obtain promising results with high computational efficiency [52,53].

The application of Bayes’ theorem together with this naive assumption makes it possible to measure the probability that the input data $x=(x_{1},x_{2},\ldots ,x_{n})$ belong to class *y* according to Equation (2).

(2) $$P\left(y|x_{1},x_{2},\ldots ,x_{n}\right)\propto P\left(y\right)\Pi_{i=1}^{n}P\left(x_{i}|y\right)$$

The class predicted by the model will be the one that maximizes this probability among all the classes present.

There are numerous and varied implementations of this method, which differ in the assumptions made about the distribution of $P(x_{i}|y)$. For this work, the following were used.

##### Gaussian Naive Bayes

The Gaussian Naive Bayes (GNB) is one of the most common implementations of NB. In this method, the assumption is that the features follow a normal distribution within each class. In this case, the assumption is that the features follow a normal distribution within each class [53,54]. Given this assumption, the conditional probability that a feature $x_{i}$ belongs to class *y* can be calculated using Equation (3).

(3) $$P\left(x_{i}|y\right)=\frac{1}{\sqrt{2\pi \sigma_{x_{i}}^{2}}}\times e^{-\frac{{\left(x_{i}-\mu_{x_{i}}\right)}^{2}}{2\sigma_{x_{i}}^{2}}}$$

where $\sigma^{2}$ is the variance and μ is the mean of the characteristic $x_{i}$ for class *y*.

##### Bernoulli Naive Bayes

The Bernoulli Naive Bayes (BNB) assumption made in this implementation is that each input feature is assumed to be a binary value [53,55]. Under this assumption, the conditional distribution $P(x_{i}|y)$, is modeled as a Bernoulli distribution, seen in Equation (4).

(4) $$P\left(x_{i}|y\right)=P\left(x_{i}=1|y\right)\times x_{i}+\left(1-P\left(x_{i}=1|y\right)\right)\left(1-x_{i}\right)$$

where $x_{i}$ is the $i^{\mathrm{th}}$ feature, and *y* is a given class.

#### 3.2.5. K-Nearest Neighbours

K-Nearest Neighbors (KNN) is a nonparametric algorithm used in supervised classification tasks. This method is based on the hypothesis that samples that are close in the feature space tend to belong to the same class. For this reason, during the training process, no parameters are adjusted, coefficients are calculated, or an explicit model is built; instead, the training data is stored [56].

When predicting the class of a new sample, it is classified according to the majority class of its K nearest neighbors. The nearest neighbors are calculated based on a distance metric. Using this method allows for the generation of nonlinear decision surfaces, which are adjusted to the class distribution of the training set. Despite being a simple but high-performance model, its computational cost increases with the volume of data, making it unmanageable in training sets with large amounts of data.

#### 3.2.6. Decision Tree

Decision Tree (DT) is a machine learning model used in both classification and regression tasks and is one of the most widely used methods [57]. These models generate a hierarchical tree-like structure, recursively dividing the training dataset. This separates the initial data set into new subsets, which are divided again until a prediction is obtained. During the inference process, when a new sample is introduced into the model, the diagram is sequentially traversed from the root node to the terminal nodes, also called leaves, which are responsible for making the prediction. At each node, a characteristic of the sample is selected and thresholds are set, according to which the sample will advance through one part or another of the hierarchical structure.

One of the main advantages of this method is its interpretability. Hierarchical tree structures allow us to understand why a sample is classified as a particular class and not another. On the other hand, a poor fit in the stopping criteria of the structure results in an overly large tree diagram, which can lead to overfitting of the model [58,59].

#### 3.2.7. Random Forest

Random Forest (RF) models are an ensemble technique, derived from the Decision Tree family of models, Section 3.2.6. These techniques are a variation of decision trees, since the result of the prediction, in both the case of regression and classification tasks, is not the result of a single decision tree, but rather the prediction of numerous trees is taken into account, resulting in the majority class (in classification tasks). The decision trees that ensemble the random forest model are trained with slightly different subsets of the data, thus preventing all DTs from yielding exactly the same result. One of the main advantages of the Random Forest technique is that it is capable of minimizing the tendency toward overfitting in DTs, a common and widespread limitation in DT applications [60].

#### 3.2.8. Gradient-Boosted Trees

Gradient-Boosted Tree (GBT) is a gradient boosting technique. In contrast to the bagging approach used by Random Forests, this machine learning method uses two different approaches: Boosting and Gradient descent [61].

Boosting is an ensemble technique in which, instead of using multiple elements during training and prediction, elements (trees) are created and added to correct the errors made by previous models and progressively improving the quality of the prediction. In this way, the system starts with weak and poorly performing classifiers, which are then improved until they become robust classifiers. The term ‘Gradient’ refers to the use of gradient descent to optimize the cost function of trained trees [62].

#### 3.2.9. Artificial Neural Networks

Artificial Neural Networks (ANN) are a widely used machine learning algorithm for classification, regression, and more tasks. These mathematical models, inspired by the structure of the human brain, are formed by the interconnection of simple units called artificial neurons. Although their performance and computational cost are largely dependent on their architecture, MultiLayer Perceptron (MLP) represent the starting point for many advanced models used in content generation using Artificial Intelligence. Despite their relative simplicity compared to more complex architectures, MLPs remain relevant for less complex tasks [63].

An MLP is composed of neurons organized in layers, where each neuron in a layer is connected to all neurons in the adjacent layers. Information processing is performed sequentially, from the input layer, which receives the characteristics of the problem, to the output layer, which generates the final prediction. Between the two are the hidden layers, whose number of neurons can vary depending on the model design. The input layer contains as many neurons as input variables, while the output layer typically has one neuron for each class to be predicted. Each neuron receives signals from neurons in the previous layer, weights them using individual coefficients (weights), sums the results together with a constant value (bias), and applies an activation function to generate its output [64].

## 4. Experimental Setup

**Figure 2:**
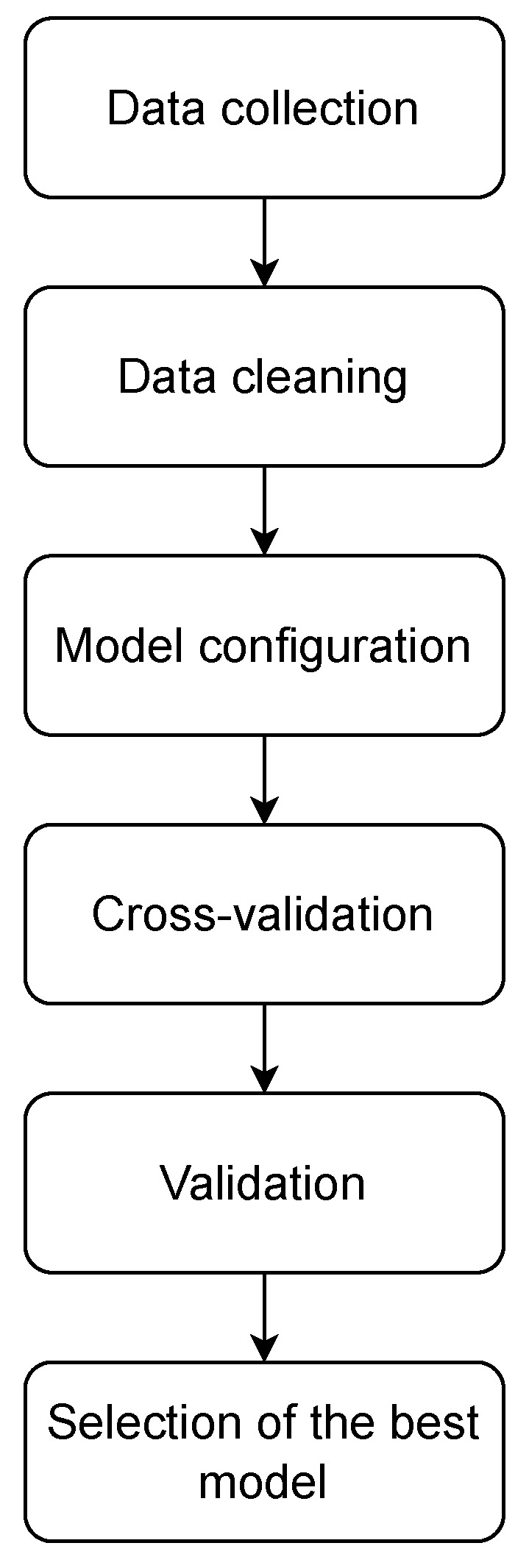
Flowchart outlining the methodology used in the research. The following steps are outlined: obtaining the dataset, preprocessing the dataset, configuring models, cross-validation, validation, and selecting the best multiclassifier.

This section presents, in detail, the procedures and experiments developed during the development of the work. Figure 2 presents a summary of the methodology, showing the different steps and the established order for obtaining a multiclassifier of cyberattacks in MQTT networks based on shallow learning techniques, which allows the detection and classification of cyberattacks at the Edge.

### 4.1. Data Cleaning

First, the three datasets mentioned in Section 4 were merged to obtain a single dataset on which it was possible to run the multi-classification algorithms. This integration was feasible since the three original datasets contained the same set of variables. Thus, a single dataset was generated that included both normal network traffic and the packets related to each of the two attacks described above. The resulting dataset contained more than 260,000 registers, with 66 predictor variables and one target variable. The distribution of classes showed a marked imbalance, with a clear predominance of normal traffic over attack records. Once the three sets were unified, the variables that had more than 90% missing values and provided hardly any information were removed. Likewise, those whose values remained constant in all records and those whose information was redundant or duplicated were discarded.

Subsequently, variables that could induce bias in the learning process were eliminated, such as network identifiers, message contents, topic names, IP and MAC addresses (both source and destination), as well as other derived variables with little statistical representativeness.

After this cleansing process, where variables with a high proportion of missing values, constant and duplicate variables, as well as variables containing information to bias the model, were eliminated, the dataset was reduced to 16 variables, of which 15 were used as predictors and one as the target variable. Non-numerical variables were coded using ordinal coding on this dataset. In particular, the target variable was recoded in numerical format with three classes: normal traffic and the two types of attack.

On the other hand, missing values, generated as a result of the high variability in message types, were imputed with an out-of-range value (−1). This allowed all records to be retained and inconsistencies in the classification algorithms to be avoided.

Finally, duplicate records were detected and removed, reducing the set to approximately 175,000 unique records. The resulting distribution confirmed the existence of a strong class imbalance, a key aspect for the subsequent modeling and evaluation phases. Specifically, the final dataset consisted of:

- **Normal**: 160,386 records.
- **DoS**: 13,076 records.
- **Intrusion**: 1898 records.

### 4.2. Model Configuration

First, to obtain the system with the best performance in detecting and classifying cyberattacks, the models for each of the previously described techniques are tuned. To do this, a sweep of values is performed on the various hyperparameters that configure the model’s operation. The different sweeps performed are indicated below.

#### 4.2.1. Linear Discriminant Analysis

The configuration of the Linear Discriminant Analysis models involved sweeping parameters such as the solver used in the optimization algorithm, the shrinkage parameter, and the absolute threshold used to consider an input value as significant.

- Singular Value Decomposition (SVD), Least Square Solution (LSQR), and Eigenvalue Decomposition (Eigen) are used as solvers.
- 0.1, 0.2, 0.3, 0.4, 0.5, 0.6, 0.7, 0.8, 0.9 are used as fixed and pre-established values, and an automatic contraction is employed, using the Ledoit–Wolf lemma.
- The values 0.0000001, 0.000001, 0.00001, 0.0001, 0.001, and 0.01 are used as the threshold for considering a singular value significant. This value is only used if the SVD solver is employed.

In total, a total of 26 LDA models were configured.

#### 4.2.2. Quadratic Discriminant Analysis

The configuration of models based on Quadratic Discriminant Analysis was achieved by modifying the value of the hyperparameter that determines regularization. The following sweep was used:

- The values 0.0, 0.1, 0.2, 0.3, 0.4, 0.5, 0.6, 0.7, 0.8, 0.9 and 1.0 were used as the regularization parameter for calculating the scaling of the Gaussian distribution, along the main axes, for each class.

This results in 11 different QDA configurations.

#### 4.2.3. Naive Bayes Bernoulli

In the Bernoulli implementation of Naive Bayes, different values of the smoothing parameter and the use of class prior probabilities are tested. The values used are listed below:

- The values $1\times 10^{-11}$, $1\times 10^{-10}$, $1\times 10^{-9}$, $1\times 10^{-8}$, $1\times 10^{-7}$, $1\times 10^{-6}$, $1\times 10^{-5}$, $1\times 10^{-4}$, $1\times 10^{-3}$, $1\times 10^{-2}$, $1\times 10^{-1}$, $1\times 10^{0}$, $1\times 10^{1}$, $1\times 10^{2}$, $1\times 10^{3}$, $1\times 10^{4}$, $1\times 10^{5}$, $1\times 10^{6}$, $1\times 10^{7}$, $1\times 10^{8}$, $1\times 10^{9}$, $1\times 10^{10}$, are tested for the smoothing parameter, α.
- The prior probabilities are adjusted based on the data (fit_prior = True) and a uniform probability distribution is used (fit_prior = False).

In total, 42 different configurations were tested.

#### 4.2.4. Naive Bayes Gauss

For this implementation of the Naive Bayes algorithm, the hyperparameter that controls the proportion of the highest variance among the features that are integrated into the process is modified. The values used are:

- The values $1\times 10^{-18}$, $1\times 10^{-17}$, $1\times 10^{-16}$, $1\times 10^{-15}$, $1\times 10^{-14}$, $1\times 10^{-13}$, $1\times 10^{-12}$, $1\times 10^{-11}$, $1\times 10^{-10}$, $1\times 10^{-9}$, $1\times 10^{-8}$, $1\times 10^{-7}$, $1\times 10^{-6}$, $1\times 10^{-5}$, $1\times 10^{-4}$, $1\times 10^{-3}$, $1\times 10^{-2}$, $1\times 10^{-1}$ and $1\times 10^{0}$ are tested for var_smoothing.

This results in 19 different models.

#### 4.2.5. K-Nearest Neighbors

For models based on K-Nearest Neighbors, the hyperparameters related to the number of neighbors used for classification and the method to determine the weight of each neighbor were modified. The values tested were:

- 5, 10, 15, 20, 25, 30, 35, 40, 45, 50, 75, 100, 125, 150, 175, 200, 250, 300, 350, 400, 450, and 500, as the number of neighbors for prediction.
- An uniform and distance-based distribution is used to assign weights to the K-nearest neighbors.

The result is 44 different models, all of them based on KNN.

#### 4.2.6. Decision Tree

The hyperparameter scan for decision tree models involved evaluating the maximum depth of the tree diagram, the function used to determine the quality of the split performed, and the split decision method. The values tested were:

- 2, 3, 4, 5, 10, 15, 20, 25, 30, 35, 40, 45, 50, 55, 60, 65, 70, 75, 80, 85, 90, 95, and 100, without explicitly limiting the depth, as maximum depths of the diagram.
- The Gini impurity and Shannon entropy functions are used as impurity calculations.
- The selection of the division is made on the basis of the best division and randomly.

A total of 144 DT-based configurations were created.

#### 4.2.7. Random Forest

The modified hyperparameters in the random forest models included the number of decision trees (estimators) used for classification, the maximum depth of each of those estimators, the criterion used for impurity calculation, and finally, the maximum number of features to consider in order to find the best split. The verified values for each hyperparameter are the following:

- 60, 70, 80, 90, 100, 110, 120, 130 and 140 trees are used to create the forest.
- The maximum tree depth used is 5, 10, 15, 20, 25, 30, 40, 50 and 60.
- The Gini impurity and Shannon entropy functions are used to measure the quality of a division.
- 10, 20, 50, 75 and 100% of the features are used to find the best split, as well as the square root (SQRT) and the base-two logarithm (LOG2) of the features.

For the RF technique, 203 models were analyzed.

#### 4.2.8. Gradient-Boosted Trees

The search for the best Gradient-Boosted Tree configuration was performed by modifying the hyperparameters of the number of trees used to obtain the model, the maximum depth of the tree diagrams, and the learning rate. The values used were:

- 30, 40, 50, 60, 70, 80, 90, 100, 110, 120, 130, 140, 150, 160, 170, 180, 190 and 200 trees to obtain the model.
- A maximum tree depth of 1, 2, 3, 4, 5, 6, 7, 8, 9, 10, 11, 12, 13, 14, 15, 16, 17, 18, 19 and 20.
- A learning rate of 0.0001, 0.001, 0.01, 0.05, 0.1, 0.5, 1, 5 and 10.

In total, 3240 different GBT models were created.

#### 4.2.9. Artificial Neural Network

The configuration of MLP-based models involved modifying the number of intermediate layers of the architecture, the number of hidden neurons (or neurons present in these intermediate layers) and the activation function of the neurons, with the exception of the three neurons in the output layer:

- The architecture was formed using 1, 3, 5, and 7 hidden layers.
- 5, 10, 15, 20, 25 and 30 hidden neurons were tested.
- Linear, ReLU, and sigmoid functions are used as the activation function of neurons.

A total of 72 different MLP architectures were obtained.

### 4.3. Cross-Validation

Once the models are created, a cross-validation process is performed to determine the performance of the different configurations. The objective of this phase is to determine the configurations with the best performance, that is, those configurations with a higher rate of correct cyberattack classification.

To do this, a 10-fold stratified cross-validation is performed on 75% of the cleaned dataset, representing a total of 131,520 records. Due to the large imbalance between classes, with an approximate ratio of 85:7:1, an oversampling technique, SMOTE, is used to transform the problem into a balanced classification problem.

This oversampling is used only on the models’ training set, balancing the classes and achieving unbiased training. However, the models are validated using original data, which were not altered by any balancing technique.

### 4.4. Validation

Once the models have been created, cross-validation has been performed, and statistical analysis has been completed, the models with the highest performance for each technique will be selected. Subsequently, each of these models will be validated with 25% of the data that have not yet been used. In this way, each model will be trained with 75% of the data (the same data that were already used during cross-validation) and validated with 25% of the data. Again, the balancing technique is used exclusively on the training dataset, using completely real data for validation.

## 5. Results

This section will discuss the results obtained during the development of the work, showing, step by step, and supported by graphs and tables, the results achieved and performing an analysis of them.

### 5.1. Cross-Validation

In this subsection, the results obtained during the stratified cross-validation process are presented, grouped by machine learning technique and algorithm. Due to the large number of models tested, the results presented refer only to the models with the most promising results.

#### 5.1.1. Linear Discriminant Analysis

The results of the stratified cross-validation process for LDA models are shown in Table 2. The most significant results are highlighted in bold.

The table shows the performance of two different configurations. Both use the same solver, LSQR, but different shrinkage values: 0.1 for one configuration and auto-calculated (based on the dataset) for the other.

In this case, a notable difference is observed in the overall performance of the models. Automatic shrinkage calculation achieves an accuracy of 78.8%, compared to 91.1% when using an explicit value, 0.1, as the shrinkage, demonstrating better generalization of this second model. Regarding the models’ performance for the normal class, setting the regularization value results in a considerable improvement in recall and F1-score, increasing from 79.2% to 95.4% and from 87.6% to 95.4%, respectively. The detection of normal MQTT communications is accurate, especially when the shrinkage hyperparameter is set to 0.1, achieving a good balance between precision and recall. This method correctly identifies a higher proportion of genuine normal communications compared to the shrinkage calculation. When analyzing the DoS class, a mixed behavior is observed. Although applying a fixed value increased precision from 40.1% to 100%, this came at the expense of a reduced recall rate, which fell from 76.7% to 45.3%. This indicates that the model has a conservative bias in predicting this type of attack, which generates numerous false negatives. Although the balance between the two metrics is 62.3%, the model’s performance is inadequate; detecting all real DoS attacks would be preferable, even at the cost of reduced precision. Detection of the Intrusion class performs well below the minimum required, with an F1-score of only 15.4%, and that is in the best-case scenario. This demonstrates the model’s inability to correctly classify the attack, resulting in disastrous accuracy and recall values, a concerning false positive rate, and even with this overprediction, only about 50% of real attacks are correctly detected.

**Figure 3:**
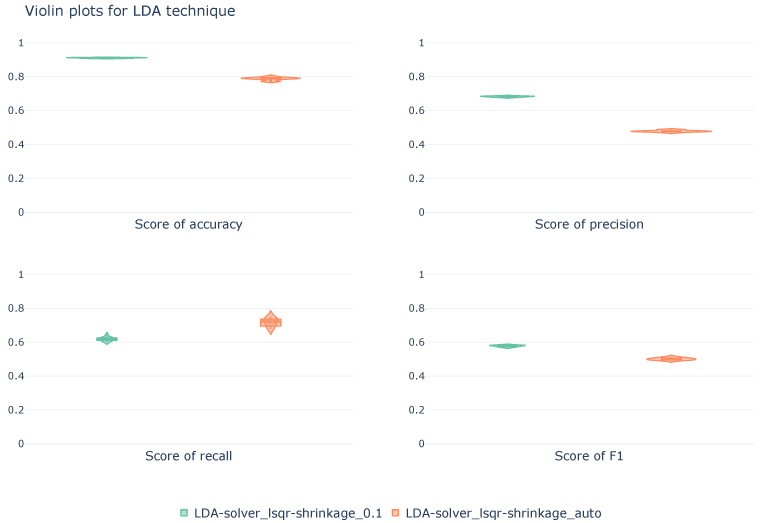
Violin diagrams illustrating the distribution of scores for the LDA technique under two different configurations. Each subplot corresponds with a unique metric. The width of each violin represents the density of values obtained across the 10k-fold cross-validation.

Figure 3 shows, in a violin plot, the mean values of the different metrics obtained during the stratified cross-validation. Unlike Table 3, these mean values are calculated from a macroscopic perspective.

The image demonstrates the superiority, in three of the four metrics, of the configuration that sets the shrinkage to 0.1 compared to the configuration that automatically determines it based on the input dataset. Automatic shrinkage configuration exhibits greater variability in all metrics, especially in recall, where the distribution is wider and has steeper tails, indicating less consistent behavior across folds. On the other hand, the fixed shrinkage configuration shows less variability, with more compact, symmetrical, and narrow distributions, demonstrating stable results.

#### 5.1.2. Quadratic Discriminant Analysis

Table 3 shows the mean values of the different metrics for each metric in the stratified cross-validation of the QDA models. The best results for each pair of metric-class are highlighted in bold.

**Table 3:** Violin diagrams illustrating the distribution of scores achieved during the cross-validation process of the QDA technique. Each color of the violin represents a different model, and there is a diagram for each metric.

Regularization of parameters: 0.0				
	acc	precision	recall	F1-score
Normal	0.435	**0.998**	0.427	0.598
DoS	0.435	**0.999**	**0.454**	**0.624**
Intrusion	0.435	0.019	**0.987**	0.036
**Regularization of parameters: 0.1**				
	acc	precision	recall	F1-score
Normal	**0.914**	0.954	**0.957**	**0.955**
DoS	**0.914**	**0.999**	0.453	**0.624**
Intrusion	**0.914**	**0.099**	0.447	**0.162**

The table shows the results of two different Quadratic Discriminant Analysis configurations, one without any regularization, and the other with a regularization of 0.1.

In general, the unregulated model exhibits extremely poor performance, with an accuracy of only 43.5%, which means that more than half of its predictions are incorrect. At the opposite extreme is the model with a regularization of 0.1, which achieves an overall accuracy of 91.4%. Analyzing the performance of the models for each individual class allows us to further refine the performance of each model. On the one hand, the detection of the normal class (MQTT communications not under attack) is adequately performed by the regularized model, which achieves an F1-score of 95.5%. However, the unregulated model, despite showing higher accuracy than the previous model, has low recall. This implies that, although the positive classifications of standard communications are correct, less than half of these actual communications are detected. When analyzing the DoS class, we observe the same phenomenon in both configurations. An accuracy of 99.9%, but a recall rate of less than 50%, implying failures in the detection of this cyberattack. Finally, performance worsens when analyzing the Intrusion class. For the detection of this cyberattack, both configurations exhibit poor accuracy and recall metrics. Neither configuration correctly detects this attack, failing to detect actual attacks or accurately predict them. The difference between accuracy and recall suggests that the model tends to overpredict the attack.

**Figure 4:**
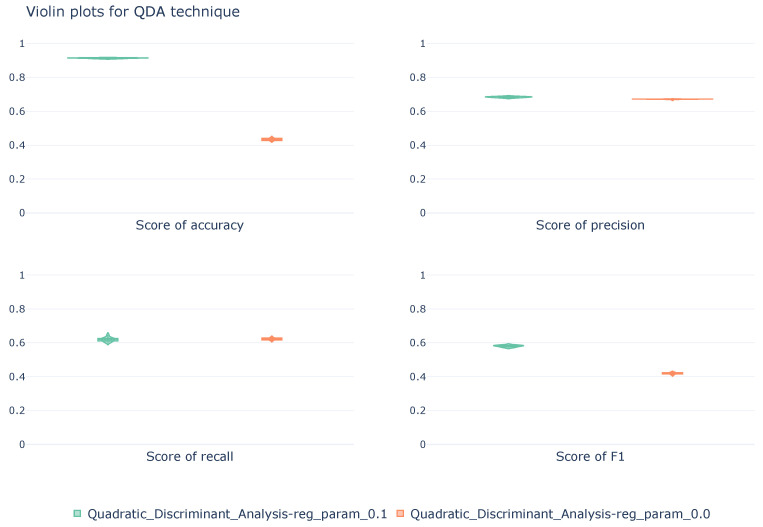
Violin diagrams illustrating the distribution of scores for the QDA technique under two different configurations. Each subplot corresponds with a unique metric. The width of each violin represents the density of values obtained across the 10k-fold cross-validation.

The violin plots, Figure 4, allow analysis of the variability of the results between folds of the stratified cross-validation for the two QDA configurations. All metrics are calculated from a macroscopic perspective. The first configuration, without regularization, has distributions centered on low values; while the second configuration, with a regularization of 0.1, shows distributions centered on higher values. However, using recall and precision metrics, it can be seen that the latter configuration exhibits wider distributions, resulting from greater inconsistency and variation in the results between folds.

#### 5.1.3. Naive Bayes

The results of the stratified cross-validation process for the Naive Bayes models are presented below. In these results, shown in Table 4, the highest values for each pair of metric-class are highlighted in bold. The table shows three different configurations: one with the Bernoulli implementation and the other two with the Gaussian implementation.

In general, all three configurations demonstrate a high capacity to correctly classify the type of MQTT communication that is being performed. Specifically, the model using the Gaussian implementation and a var_smoothing of 1 achieves the best accuracy, at 94.9%. The models exhibit robust performance with respect to the Normal class, with an accuracy close to 95.4% and a recall greater than 95.6% in Bernoulli, increasing to 99.8% in the Gaussian implementation. The F1-score, which reaches its maximum (97.5%) in Gaussian implementation, reflects a good balance between precision and recall. The configurations, when dealing with the DoS class, exhibit more variable behavior. In Gaussian implementation, the recall remains low, at 45.4%, while the accuracy ranges from 75.9% to 99.9%, depending on the parameter var_smoothing. It is evident how this parameter affects the false positive rate, which improves as the parameter increases. However, recall remains at the same value, indicating poor detection of real DoS attacks, detecting less than 50% of actual attacks. The parameter var_smoothing only improved the quality of the positive predictions, reducing the number of false positives but maintaining the false negatives. The F1-score improves slightly in the second configuration, reaching 62.4%, but this is still limited. In the case of Bernoulli implementation, the accuracy reaches 58.5%, but the recall remains at 45.4%, demonstrating poor performance in DoS detection.

**Figure 5:**
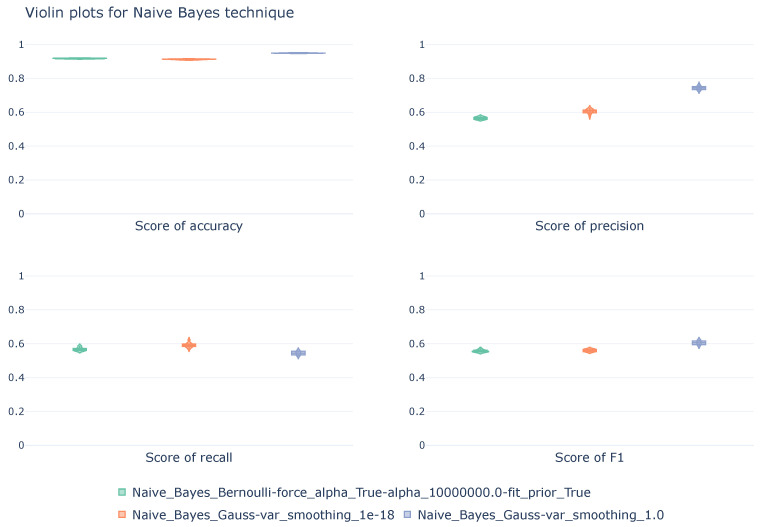
Violin diagrams illustrating the distribution of scores for the NB technique under three different configurations. Each subplot corresponds with a unique metric. The width of each violin represents the density of values obtained across the 10k-fold cross-validation.

Figure 5 represents the distribution of scores, calculated using a macro average, throughout the cross-validation process. The violin plots, representing the three Naive Bayes configurations in Table 4, show similar performance among the three models, except for the precision metric, where the Gaussian implementation of the model with a var_smoothing factor of 1 clearly stands out. On the other hand, the distribution of scores is quite concentrated, demonstrating the robustness of the models against different data distributions.

#### 5.1.4. K-Nearest Neighbors

Next, in this section, the results of the cross-validation process for the KNN configurations are presented and discussed. Table 5 shows the average values of the metrics obtained by the best-performing models in stratified cross-validation. The best values for each of the classes and metrics are highlighted in bold.

Regarding the model configuration, the weight assignment based on neighbor distance is the distribution present in the three models, and again, all three configurations use a moderate number of neighbors: 5, 10, and 20.

All three models achieve high performance in correctly classifying records, with accuracies greater than 98%. Analyzing the metrics individually for each class, it can be seen that the Normal and DoS classes achieve high performance in all metrics: precision, recall, and F1-score, regardless of the model. Although all models perform similarly, regarding the classification of the Normal class, the model that uses fiveneighbors stands out; compared to the one that uses 20 neighbors, it excels in the classification of DoS attacks, obtaining the best metrics for these classes. However, on the other hand, the classification of the Intrusion class does not achieve such excellent performance, seeing a decrease in precision for all three configurations. The recall metric continues to perform well, albeit with a slight decrease. Therefore, attacks are being detected, but this cyberattack is being overclassified, implying a bias in the models toward predicting this type of attack.

**Figure 6:**
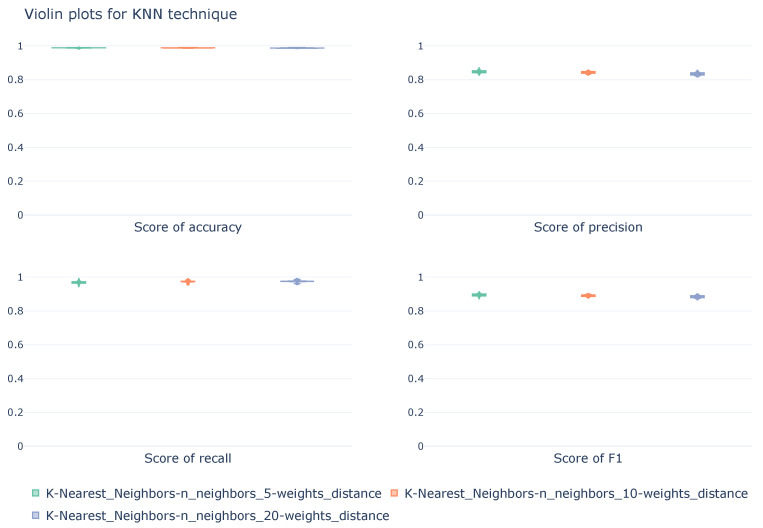
Violin diagrams illustrating the distribution of scores for the KNN technique under three different configurations. Each subplot corresponds with a unique metric. The width of each violin represents the density of values obtained across the 10k-fold cross-validation.

Figure 6 shows, in a violin graph, the distribution of accuracy, precision, recall, and F1-score metrics for the three models during the cross-validation process, calculated from a macroscopic perspective. These images show how all models have low viability, with narrow violins, indicating the stability of the models across the different folds that make up the cross-validation.

#### 5.1.5. Decision Tree

Table 6 below shows the average values of the metrics obtained during cross-validation of the Decision Tree-based configurations. The best values for each of the calculations are in bold. The best values for each calculation are shown in bold.

The table presents three DT models that differ in the maximum depth of the diagram, impurity criterion, and splitting strategy. The three trees use different depths (20, 25, and 30) while sharing, in pairs, the impurity criterion and the splitting strategy.

From a general perspective, the three models exhibit identical behavior. Regardless of maximum depth, impurity criterion, and splitting strategy, an accuracy of 99.2% is achieved, indicating excellent overall communication detection and classification quality. Analyzing the performance of each configuration for individual classes provides a more detailed understanding of each model’s behavior. When considering normal communications, which are not under attack, the three models demonstrate excellent and similar performance. All three models achieve precision and recall rates greater than 99%, which means that the detection of these communications is highly effective. The DoS class again demonstrates commendable performance across all three configurations, but with a greater difference between models. For detecting this type of attack, the model with a maximum diagram depth of 25 nodes stands out, achieving the best score across all three metrics, although all three configurations deliver similar performance. The Intrusion class shows a change compared to the previous classes. In this case, although the detection of real attacks is adequate and with very high detection rates, exceeding 93%, there is a reduction in the quality of the prediction, with an increased number of false positives compared to previous classes. This increase in prediction failures is reflected in the precision metric, which drops to 71.4% in the best case, corresponding to the model with a maximum diagram depth of 35 nodes, the Gini impurity criterion, and a random splitting strategy. In any case, although the false positive rate increased, the balance between precision and recall (F1-score) remains adequate, at around 80% in all three cases. The model with the best F1-score uses 20 nodes as its maximum depth, entropy as the impurity criterion, and a splitting strategy based on the best split, although it does not achieve the best results in either precision or recall. With a slightly lower F1-score (0.3 points lower), the model with a maximum depth of 35 nodes, Gini coefficient as impurity criterion, and a random splitting strategy exhibits the second-highest recall (0.977) and the highest precision.

**Figure 7:**
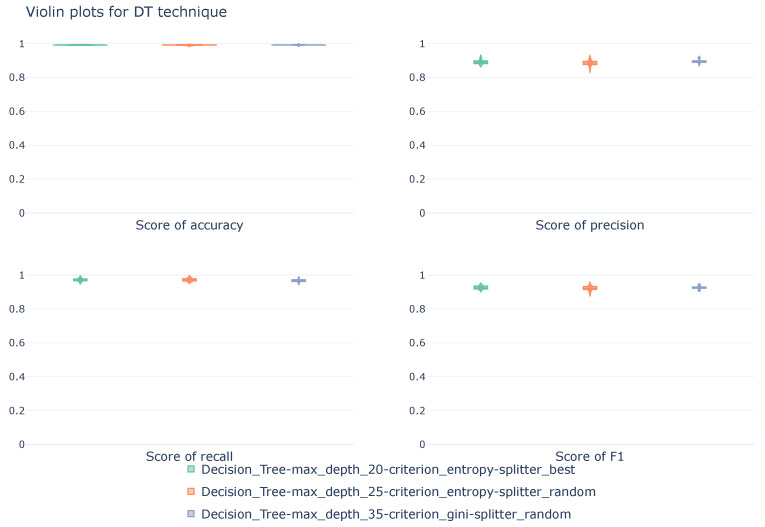
Violin diagrams illustrating the distribution of scores for the DT technique under three different configurations. Each subplot corresponds with a unique metric. The width of each violin represents the density of values obtained across the 10k-fold cross-validation.

Using another representation, shown in Figure 7, it can be seen how the models maintain similar performance across all four metrics. The scores, calculated using a macro approach, exhibit a close distribution throughout the cross-validation process, demonstrating the robustness of the configurations.

#### 5.1.6. Random Forest

Table 7 shows the average results of three Random Forest configurations obtained by stratified cross-validation. The highest results, calculated for each pair of metric-class, are highlighted in bold.

The three configurations shown in the table above share two of the four adjusted hyperparameters: the impurity calculation criterion, which is set to Entropy; and the maximum number of features used by the models, which is half the number of features in the original dataset. The three configurations differ in the number of decision trees used for prediction, with values of 70 and 130; and in the maximum depth of the tree diagrams, with values of 15 and 20 nodes. From an overall perspective, all three configurations demonstrate excellent performance. Accuracy is 99.2% for all three models, indicating a high classification capacity. Analyzing the performance and behavior of the models for each class reveals some differences. Evaluating the models’ ability to correctly detect normal MQTT communications, all three models achieve outstanding performance, with accuracy and recall of 99.8% and 99.4%, respectively. For this specific class, all three configurations exhibit the same behavior, showing no differences in the average value. The DoS class, like the Normal class, demonstrates very high performance. In this case, there is a slight variation in the results obtained when considering the parameter configuration, although this variation is minimal. The F1-score values range from 98.4% to 98.8%, depending on the model, indicating a good balance between precision and recall. However, when analyzing the performance of the configurations for the Intrusion attack, a reduction in model performance is observed. Although recall remains high, at 97.1% for models with a maximum depth of 15 nodes, accuracy drops to 66.9% for the model with 130 estimators and a maximum depth of 15 nodes. This indicates that, while most real Intrusion attacks are detected, the models generate numerous false positives, thus reducing accuracy. Even with this performance reduction, the balance between accuracy and recall is high, 81.9% for the model with the highest maximum depth in its diagrams, which achieves the best accuracy value, at the cost of slightly lower recall compared to the other models.

Reviewing the results, it can be seen that the number of estimators has a minimal impact on model performance, indicating that the configuration with 130 trees does not offer a significant benefit compared to the configuration with fewer estimators (70). Increasing the maximum depth of the diagrams did have a greater impact, modifying the balance between precision and recall in the Intrusion class.

**Figure 8:**
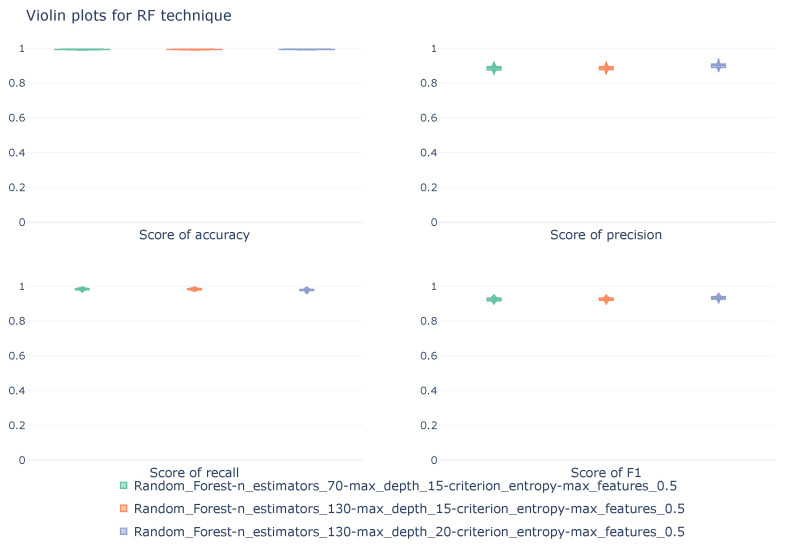
Violin diagrams illustrating the distribution of scores for the RF technique under three different configurations. Each subplot corresponds with a unique metric. The width of each violin represents the density of values obtained across the 10k-fold cross-validation.

The violin diagrams, Figure 8, represent the distribution of the metric scores for the four configurations listed in Table 7. These scores, calculated using a macro average, do not vary significantly between the different folds of the cross-validation, as represented by the short tails of the violins.

#### 5.1.7. Gradient-Boosted Tree

Table 8 below shows the average results of the metrics for each of the three classes during the stratified cross-validation process for four different Gradient-Boosted Tree models. The best results for each pair of metric-class appear in bold.

The four configurations listed in the table above vary in the number of estimators, the maximum depth of the diagrams, and the learning rate.

All configurations, regardless of their hyperparameter configuration, achieve high accuracy values, above 90%, demonstrating a high detection capacity of cyberattacks on IoT communications in MQTT. Analyzing the performance of the models for each individual class, it is observed that the standard communications classification, without any attacks, has outstanding performance. All models achieve a good balance between precision and recall, with an F1-score of 0.995%. However, the model with the highest recall, that is, the model that detects the highest percentage of real normal communications, is the model with 180 estimators, a maximum depth of 11 and a learning rate of 0.1. Nevertheless, the model with the highest recall (that is, the model that detects the highest percentage of normal communications) is the one with 180 estimators, a maximum depth of 11, and a learning rate of 0.1. However, the performance of the different configurations for detecting DoS cyberattacks is outstanding. The model using 60 estimators, a maximum diagram depth of 7, and a learning rate of 0.1 achieves the best balance, the F1-score, between accuracy and recall; while more complex models, such as the one with 180 estimators, exhibit the best recall, detecting a greater number of genuine DoS attacks.

Classification performance decreases when considering communications under an intrusion. This deterioration is widespread across all four models, which show an increase in the number of false positives in the prediction of these attacks. This is reflected in the recall rate, which drops to 68.2% in the best-case scenario. This model, which uses 180 estimators, achieves an F1-score of 79.7% for this type of attack, considerably below that 99.5% and 97.9% for normal communications and DoS attacks, respectively.

**Figure 9:**
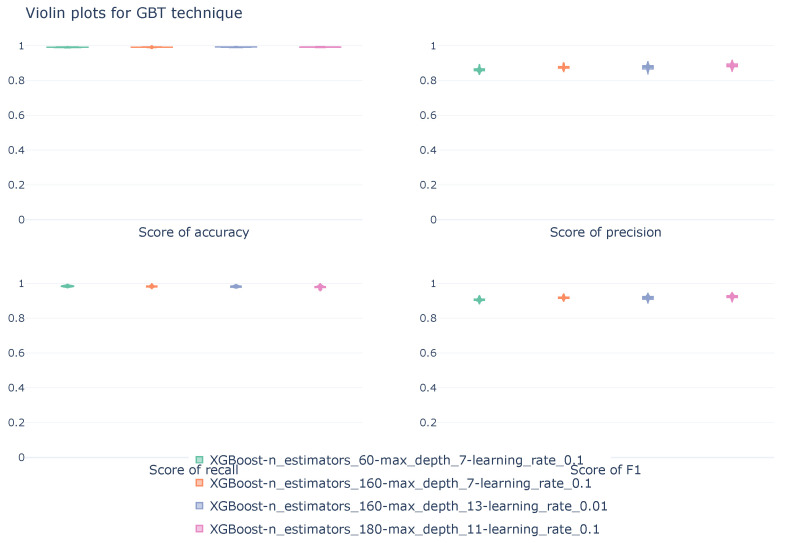
Violin diagrams illustrating the distribution of scores for the DT technique under four different configurations. Each subplot corresponds with a unique metric. The width of each violin represents the density of values obtained across the 10k-fold cross-validation.

The violin plots, represented in Figure 9, show the distribution of scores obtained during cross-validation for the GBT configurations listed in Table 9. All metrics are calculated from a macro perspective.

The violin plot demonstrates the robustness of the four configurations throughout the 10-fold cross-validation process. The violins, for each metric and model, exhibit a short tail, representing a concentrated distribution of scores, which confirms the stability of the models. When comparing the models, considerable similarity is observed between them, with slight variations in the vertical position of the violin, which corresponds to better performance on that metric.

#### 5.1.8. Artificial Neural Networks

The average results of the metrics for each class during the stratified cross-validation process of the MLP-based models are shown in Table 9. The best values for each pair of metric-class are highlighted in bold.

**Table 9:** Average MLP values during cross-validation.

Hidden layers: 5, Hidden neurons: 20, Dropout: 0, Function activation: ReLU				
	acc	precision	recall	F1-score
Normal	**0.981**	**0.998**	0.982	0.990
DoS	**0.981**	**0.974**	**0.970**	**0.972**
Intrusion	**0.981**	0.398	0.937	0.556
**Hidden layers: 5, Hidden neurons: 25, Dropout: 0, Function activation: ReLU**				
	acc	precision	recall	F1-score
Normal	**0.981**	**0.998**	**0.983**	**0.991**
DoS	**0.981**	0.966	0.961	0.963
Intrusion	**0.981**	**0.416**	**0.954**	**0.577**

The table shows two MLP configurations that differ only in the number of hidden layers. One model uses 20 hidden layers, while the second model increases the number of hidden layers to 25. The remaining parameters (number of hidden neurons, dropout, and activation function) remain constant at five neurons per hidden layer, no dropout regularization, and ReLU, respectively.

Both models exhibit a high overall classification rate, reaching 98.1% accuracy, correctly detecting the cyberattack present in communications in most instances, regardless of the architecture. However, when examining the remaining metrics by performing a class-by-class analysis, certain differences in the models’ behavior are detected. Normal communications classification demonstrates outstanding performance, with 99.8% accuracy and recall rates exceeding 98% in both configurations. Increasing the number of hidden layers had minimal impact on model performance, increasing recall and F1-score by only 0.1% compared to the simpler model. A similar phenomenon occurs when analyzing the performance of multiclassifiers for DoS attacks. Both configurations show excellent performance, although slightly lower than the normal class, in detecting DoS attacks, reaching accuracy and recall values exceeding 96%. However, in contrast to the previous case, here increasing the complexity of the neural network architecture, by increasing the number of layers, resulted in a detriment to all three metrics. Finally, analyzing the performance of the models for the Intrusion class, the decline in classification quality is evident. Although recall remains high, exceeding 95% for the model with a more complex architecture, the accuracy value plummeted to 41.6% for the same configuration; while the model correctly detects and classifies real Intrusion attacks, it tends to overdetect this attack, generating many false positives that reduce the accuracy metric. When comparing the two configurations, an increase in the number of layers led to improvements in accuracy, recall, and F1-score.

**Figure 10:**
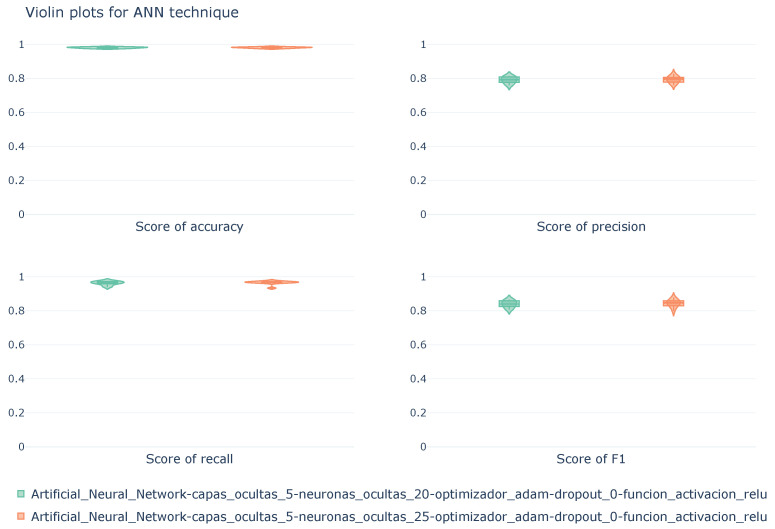
Violin diagrams illustrating the distribution of scores for the RF technique under four different configurations. Each subplot corresponds with a unique metric. The width of each violin represents the density of values obtained across the 10k-fold cross-validation.

Figure 10 shows, in a violin plot, the distribution of scores obtained during cross-validation for the two ANN configurations discussed previously. The scores shown were calculated using macro averaging.

The violin plots reflect the similarity in the metrics, which was also observed in Table 9. Both configurations exhibit similar performance, with little difference between them and high stability in each of the metrics throughout the 10 folds of the cross-validation.

### 5.2. Validation

The following subsection presents the results obtained during the validation process for the configurations described in the previous subsection. For the validation process, 75% of the data (the same data used for stratified cross-validation) was used to train the models, and the remaining 25% was used to validate the models. The oversampling process, performed using SMOTE, was carried out only during the training phase. As in the previous section, tables and graphs will be used to present the results obtained in this process.

In summary, these are the configurations selected to represent each of the machine learning techniques and algorithms:

- Linear Discriminant Analysis: LSQR solver and a shrinkage factor of 0.1
- Quadratic Discriminant Analysis: 0.1 as regularization parameter
- Naive Bayes: Gaussian implementation and a variable smoothing of 1
- K-Nearest Neighbors: use of five neighbors to make the prediction and a weight assignment based on distance.
- Decision Tree: Maximum depth of 35, Gini impurity criterion and a random splitter strategy
- Random Forest: 130 estimators, maximum depth of 20, Shannon entropy criterion and 50% of features
- Gradient-Boosted Trees: 180 estimators, a maximum diagram depth of 11 nodes, and a learning rate of 0.1
- Artificial Neural Network: five hidden layers, 25 hidden neurons and ReLU activation function

Table 10 collects the metrics, obtained during the cross-validation process, of the different techniques. From the table above, it can be seen that the performance of the models remained unchanged compared to that obtained during the stratified cross-validation process. The results obtained demonstrate that there is no overfitting in the models, allowing for a specific generalization to be made in specific models.

When analyzing model performance, the Random Forest technique stands out for its high accuracy (99.3%), demonstrating a strong ability to correctly classify ongoing communications. The Decision Tree and Gradient-Boosted Tree techniques, although 0.1 points lower, also show a strong capacity to correctly detect communications. Subsequently, though at a different level, K-Nearest Neighbors and Artificial Neural Network generally perform well. Examining the performance of the techniques by class, Random Forest again excels, achieving the highest values in all three metrics for the Normal class. In terms of precision, it achieves 99.8%, tied with Decision Tree and Artificial Neural Network, indicating a high true positive rate. In addition to correctly predicting positive results for normal communications, it detects the vast majority of actual normal communications, with a recall rate of 99.4%, surpassing any other model. Finally, the balance between these two metrics is high, with an F1-score of 99.6%, tied to Decision Tree. Decision Tree’s performance is virtually identical to that of Random Forest, with a recall rate only 0.1% lower. The Gradient-Boosted Tree model exhibits similar behavior to Random Forest, accurately classifying normal communications. On the other hand, regarding the DoS class, Linear Discriminant Analysis and Random Forest stand out as the best-performing techniques. The Linear Discriminant Analysis model demonstrates the highest precision, at 100%, indicating that all positive predictions were correct. However, the false negative rate for this model is high, with a recall of 45.7%. Random Forest, although with a lower precision of 98.5%, presents the highest recall and F1-score values of all models. Both metrics show a value of 98.6%, tied with Decision Tree in the case of recall. Random Forest’s performance in the DoS class, as in the normal class, is excellent, correctly classifying the majority of DoS samples. In this case, Gradient-Boosted Trees shows a similar performance, with worse precision, 1.3 points lower, indicating that the positive predictions made are incorrect in a greater proportion. Finally, when considering the Intrusion class, a decline in performance is observed across all techniques. None of them maintains the performance achieved by other classes within the Intrusion class. The generalization of this class is considerably worse. The best-performing techniques are Decision Tree, with an accuracy of 72.3%, Gradient-Boosted Trees, with a recall of 95.8%, and Random Forest, with an F1-score of 81.4%. Models generally exhibit a high recall rate, which means that they correctly detect real intrusion attacks; however, they also have a high false positive rate, which reduces accuracy. Nevertheless, although the performance of the models is poorer and the detection of these types of attack is not as accurate as in previous classes, the results are still acceptable, with a balance between precision and recall of 81.4%. For the Random Forest model, the recall is 94.9% compared to 71.2% precision. Despite the model’s tendency to overpredict this type of attack, its performance is adequate.

The results shown in Table 10, with the best results for each metric-class pair highlighted in bold, which collect the metrics of the validation process of the best configuration of each of the Machine Learning techniques selected to develop a shallow learning-based multiclassifier for MQTT communications, together with the review and analysis of these in the previous paragraph, highlight Random Forest as the best algorithm for this purpose. Of all the validated models, a total of eight different algorithms, Random Forest exhibits the highest accuracy metric at 99.3% (0.1% higher than the next highest algorithm), making RF the technique with the highest absolute number of correct detections. However, given the significant imbalance in the problem, additional metrics are necessary to evaluate. Evaluating the classes independently reveals that RF achieves the highest F1-score in each of the three classes to be detected, indicating that it is the model with the best balance between precision and recall. This balance is 99.6%, 98.6%, and 81.4% for the Normal, Dos and Intrusion classes, respectively. A strong overall classification system, along with the best balance between accuracy and recall, positions Random Forest as the leading method for multiclassifying cyberattacks on MQTT networks. Its classification performance for both normal and low-doS communications is excellent, achieving the highest accuracy for the Normal class and the best recall for DoS. It is only surpassed by Naive Bayes in correctly classifying real-world normal data (by 0.5%) and by Linear Discriminant Analysis in accurately predicting DoS attacks (by 1.5%). Performance is compromised in the correct detection of the Intrusion class. Random Forest (along with the other models) does not exhibit the generalization capabilities present in the first two classes. Thus, precision drops to 71.2%, and recall, although higher, also drops to 94.9%. The sensivity of the model remains high, an aspect that is especially interesting in the cybersecurity section, as it ensures the correct detection of most real attacks in exchange for a higher rate of false positives. Although there are models with better metrics, such as Decision Tree with accuracy and Gradient-Boosted Tree with recall, Random Forest again shows the highest F1-score, with a suitable balance between both metrics, a balance not achieved by either of the previously mentioned methods.

**Figure 11:**
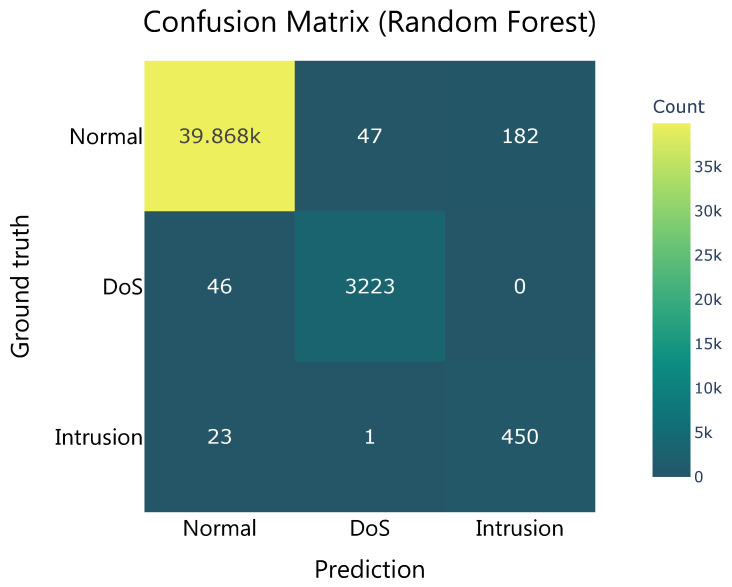
Confusion matrix of the Random Forest classifier during validation, illustrating prediction performance across Normal, Dos and Intrussion clasess.

Examining the confusion matrix, Figure 11 yields several conclusions. First, the model’s detection capabilities for normal communications and DoS attacks are noteworthy. Of the 39,937 predictions made for normal communications, 39,868 were correct, while of the 3271 DoS predictions, 3223 were accurate. Similarly, of the 40,097 actual samples of normal communications, 39,868 were correctly classified, while in the case of DoS attacks, 3223 out of 3269 attacks were detected. However, the drop in detection performance is evident in the Intrusion class. Analyzing the distribution of the model’s predictions, it can be seen that the model correctly classified 182 samples belonging to the Normal class as Intrusions, compared to 450 correct predictions for this class. Of the 474 real-world samples, the model classified 23 as normal communications, 1 as a DoS attack, and the remaining 450 as intrusions. The model tends to overpredict the Intrusion category, erroneously assigning this attack to Normal communications.

This methodological approach prioritizes the detection of structural vulnerabilities over the particularities of any specific implementation, granting the results broad applicability across diverse domains. By focusing on inherent communication behaviors, the proposed solution is positioned as a versatile tool for various technological ecosystems sharing these foundations, ensuring the preservation of its integrity and relevance in the face of the heterogeneity and noise characteristic of large-scale industrial deployments.

## 6. Conclusions and Future Works

This paper proposes an approach for detecting and classifying cyberattacks on MQTT networks. This system is based on shallow learning techniques, allowing its deployment on typical IoT devices with limited resources for monitoring network communications. A dataset was developed on an MQTT network subjected to various attacks, including Denial of Service and Intrusion, in addition to normal communications. The results obtained using Random Forest demonstrate that the proposed system functions correctly, with an accuracy exceeding 99% and an F1-score greater than 80% in detecting Intrusion attacks. Identifying the type of attack improves security and attack response management, enabling decisions to be made based on the specific attack taking place.

Future research proposes expanding the scope of the cyberattack detector and classifier to include more IoT communication protocols, such as CoAP. More attacks could also be integrated, increasing the degree of system coverage, including, for example, Man-in-the-Middle attacks.

## Figures and Tables

**Table 1:** Summary of the dataset organization by attack scenario.

CSV File	Attack Type	Frames	Normal Frames	Attack Frames
DoS.csv	Denial of Service	94,625	49,112	45,513
Intrusion.csv	Unauthorized Client	80,893	78,995	1898

**Table 2:** Average LDA values during cross-validation.

Solver: LSQR, Shrinkage: Auto				
	acc	precision	recall	F1-score
Normal	0.788	**0.980**	0.792	0.876
DoS	0.788	0.401	**0.767**	0.527
Intrusion	0.788	0.054	**0.591**	0.099
**Solver: LSQR, Shrinkage: 0.1**				
	acc	precision	recall	F1-score
Normal	**0.911**	0.954	**0.954**	**0.954**
DoS	**0.911**	**1.000**	0.453	**0.623**
Intrusion	**0.911**	**0.093**	0.447	**0.154**

**Table 4:** Average NB values during cross-validation.

Implementation: Bernoulli, α: 1 × 10^7^, fit_prior: True				
	acc	precision	recall	F1-score
Normal	0.917	**0.954**	0.962	0.958
DoS	0.917	0.585	**0.454**	0.511
Intrusion	0.917	0.154	0.284	0.199
Implementation: Gaussian, var_smoothing: 1 × 10^−18^				
	acc	precision	recall	F1-score
Normal	0.912	**0.954**	0.956	0.955
DoS	0.912	0.759	**0.454**	0.568
Intrusion	0.912	0.101	**0.362**	0.157
**Implementation: Gaussian, var_smoothing: 1**				
	acc	precision	recall	F1-score
Normal	**0.949**	0.952	**0.998**	**0.975**
DoS	**0.949**	**0.999**	**0.454**	**0.624**
Intrusion	**0.949**	**0.279**	0.180	**0.219**

**Table 5:** Average KNN values during cross-validation.

**# of neighbors: 5, Weight distribution: Distance-based**				
	acc	precision	recall	F1-score
Normal	**0.988**	**0.998**	**0.989**	**0.994**
DoS	**0.988**	0.965	**0.985**	0.975
Intrusion	**0.988**	**0.579**	0.931	**0.714**
# of neighbors: 10, Weight distribution: Distance-based				
	acc	precision	recall	F1-score
Normal	**0.988**	**0.998**	**0.989**	0.993
DoS	**0.988**	0.970	**0.985**	0.978
Intrusion	**0.988**	0.556	0.947	0.700
# of neighbors: 20, Weight distribution: Distance-based				
	acc	precision	recall	F1-score
Normal	0.987	**0.998**	0.988	0.993
DoS	0.987	**0.976**	**0.985**	**0.981**
Intrusion	0.987	0.525	**0.953**	0.677

**Table 6:** Average DT values during cross-validation.

Max. depth: 20, Criterion: Entropy, Splitter: Best				
	acc	precision	recall	F1-score
Normal	**0.992**	**0.998**	**0.993**	**0.996**
DoS	**0.992**	0.970	**0.985**	0.978
Intrusion	**0.992**	0.712	0.938	**0.809**
Max. depth: 25, Criterion: Entropy, Splitter: Random				
	acc	precision	recall	F1-score
Normal	**0.992**	**0.998**	**0.993**	0.995
DoS	**0.992**	**0.973**	**0.985**	**0.979**
Intrusion	**0.992**	0.685	**0.979**	0.792
**Max. depth: 35, Criterion: Gini, Splitter: Random**				
	acc	precision	recall	F1-score
Normal	**0.992**	**0.998**	**0.993**	**0.996**
DoS	**0.992**	0.971	0.984	0.977
Intrusion	**0.992**	**0.714**	0.977	0.806

**Table 7:** Average RF values during cross-validation.

# of estimators: 70, Max. depth: 15, Criterion: Entropy, # max. of features: 0.5				
	acc	precision	recall	F1-score
Normal	**0.993**	**0.998**	**0.994**	**0.996**
DoS	**0.993**	**0.992**	0.984	**0.988**
Intrusion	**0.993**	0.668	**0.971**	0.791
# of estimators: 130, Max. depth: 15, Criterion: Entropy, # max. of features: 0.5				
	acc	precision	recall	F1-score
Normal	**0.993**	**0.998**	**0.994**	**0.996**
DoS	**0.993**	**0.992**	0.984	**0.988**
Intrusion	**0.993**	0.669	**0.971**	0.792
**# of estimators: 130, Max. depth: 20, Criterion: Entropy, # max. of features: 0.5**				
	acc	precision	recall	F1-score
Normal	**0.993**	**0.998**	**0.994**	**0.996**
DoS	**0.993**	0.984	**0.985**	0.984
Intrusion	**0.993**	**0.719**	0.953	**0.819**

**Table 8:** Average GBT values during cross-validation.

# of estimators: 60, Max. depth: 7, Learning rate: 0.1				
	acc	precision	recall	F1-score
Normal	0.991	0.998	0.991	**0.995**
DoS	0.991	**0.994**	0.982	**0.988**
Intrusion	0.991	0.589	**0.980**	0.736
# of estimators: 160, Max. depth: 7, Learning rate: 0.1				
	acc	precision	recall	F1-score
Normal	0.991	**0.999**	0.992	**0.995**
DoS	0.991	0.978	**0.985**	0.982
Intrusion	0.991	0.651	0.973	0.780
# of estimators: 160, Max. depth: 13, Learning rate: 0.01				
	acc	precision	recall	F1-score
Normal	**0.992**	0.998	0.992	**0.995**
DoS	**0.992**	0.985	0.984	0.984
Intrusion	**0.992**	0.644	0.972	0.774
**# of estimators: 180, Max. depth: 11, Learning rate: 0.1**				
	acc	precision	recall	F1-score
Normal	**0.992**	0.998	**0.993**	**0.995**
DoS	**0.992**	0.974	**0.985**	0.979
Intrusion	**0.992**	**0.682**	0.961	**0.797**

**Table 10:** Metrics obtained in the validation process.

Linear Discriminant Analysis				
	acc	precision	recall	F1-score
Normal	0.912	0.955	0.955	0.955
DoS	0.912	**1.000**	0.457	0.627
Intrusion	0.912	0.098	0.462	0.161
Quadratic Discriminant Analysis				
	acc	precision	recall	F1-score
Normal	0.915	0.955	0.958	0.956
DoS	0.915	0.999	0.459	0.629
Intrusion	0.915	0.103	0.462	0.169
Naive Bayes				
	acc	precision	recall	F1-score
Normal	0.949	0.952	**0.999**	0.975
DoS	0.949	0.999	0.459	0.629
Intrusion	0.949	0.277	0.175	0.214
K-Nearest Neighbors				
	acc	precision	recall	F1-score
Normal	0.989	**0.998**	0.990	0.994
DoS	0.989	0.965	0.984	0.974
Intrusion	0.989	0.592	0.937	0.725
Decision Tree				
	acc	precision	recall	F1-score
Normal	0.992	**0.998**	0.993	**0.996**
DoS	0.992	0.968	**0.986**	0.977
Intrusion	0.992	**0.723**	0.905	0.804
Random Forest				
	acc	precision	recall	F1-score
Normal	**0.993**	**0.998**	0.994	**0.996**
DoS	**0.993**	0.985	**0.986**	**0.986**
Intrusion	**0.993**	0.712	0.949	**0.814**
Gradient-Boosted Tree				
	acc	precision	recall	F1-score
Normal	0.992	**0.998**	0.992	0.995
DoS	0.992	0.972	**0.986**	0.979
Intrusion	0.992	0.683	**0.958**	0.797
Artificial Neural Network				
	acc	precision	recall	F1-score
Normal	0.982	**0.998**	0.984	0.991
DoS	0.982	0.968	0.957	0.963
Intrusion	0.982	0.419	0.956	0.583

## Data Availability

The complete dataset used in this work is publicly available on Figshare (https://figshare.com/s/7421593360b8fdccb306, accessed on 22 November 2025).

## Abbreviations

The following abbreviations are used in this manuscript:

ACC	Accuracy
ANN	Artificial Neural Network
BLE	Bluetooth Low Energy
BNB	Bayesian Naive Bayes
CSV	Comma Separated Values
CoAP	Constrained Application Protocol
DDoS	Distributed Denial-of-Service
DDS	Data Distribution Service
DNS	Domain Name System
DoS	Denial-of-Service
DT	Decision Tree
GBT	Gradient-Boosted Trees
GNB	Gaussian Naive Bayes
IDS	Intrusion Detection System
IoT	Internet of Things
IP	Internet Protocol
KNN	K-Nearest Neighbors
LAN	Local Area Network
LDA	Linear Discriminant Analysis
LSQR	Least Square Solution
MAC	Media Access Control
MLP	MultiLayer Perceptron
MQTT	Message Queuing Telemetry Transport
NB	Naive Bayes
QDA	Quadratic Discriminant Analysis
QoS	Quality of Service
RF	Random Forest
SMOTE	Synthetic Minority Over-sampling Technique
SQRT	Square Root
SVD	Singular Value Decomposition
WLAN	Wide Local Area Network
XMPP	eXtensible Messaging and Presence Protocol

## References

[1] Malik P.K., Sharma R., Singh R., Gehlot A., Satapathy S.C., Alnumay W.S. (2021). Industrial Internet of Things and its applications in industry 4.0: State of the art. Comput. Commun..

[2] Teoh Y.K., Gill S.S., Parlikad A.K. (2021). IoT and fog computing based predictive maintenance model for effective asset management in industry 4.0 using machine learning. IEEE Internet Things J..

[3] Nauman A., Qadri Y.A., Amjad M., Zikria Y.B., Afzal M.K., Kim S.W. (2020). Multimedia internet of things: A comprehensive survey. IEEE Access.

[4] Krejčí R., Hujňák O., Švepeš M. Security survey of the IoT wireless protocols. Proceedings of the 2017 25th Telecommunications Forum, TELFOR 2017-Proceedings.

[5] Sharma C., Gondhi N.K. Communication protocol stack for constrained IoT systems. Proceedings of the 2018 3rd International Conference On Internet of Things: Smart Innovation and Usages, IoT-SIU 2018.

[6] Statista (2025). Petroc Taylor. Umber of IoT Connections Worldwide 2022–2034. https://www.statista.com/statistics/1183457/iot-connected-devices-worldwide/.

[7] Mahbub M. (2020). Progressive researches on IoT security: An exhaustive analysis from the perspective of protocols, vulnerabilities, and preemptive architectonics. J. Netw. Comput. Appl..

[8] Khalid M.H., Murtaza M., Habbal M. Study of security and privacy issues in internet of things. Proceedings of the CITISIA 2020-IEEE Conference on Innovative Technologies in Intelligent Systems and Industrial Applications.

[9] Pallavi S., Narayanan V.A. An overview of practical attacks on BLE based IOT devices and their security. Proceedings of the 2019 5th International Conference on Advanced Computing and Communication Systems (ICACCS).

[10] Oconnor T.J., Jessee D., Campos D. Through the Spyglass: Towards IoT companion app Man-in-the-Middle attacks. Proceedings of the CSET’21: Cyber Security Experimentation and Test Workshop.

[11] Al Enany M.O., Harb H.M., Attiya G. A comparative analysis of MQTT and IoT application protocols. Proceedings of the ICEEM 2021-2nd IEEE International Conference on Electronic Engineering.

[12] Chen F., Huo Y., Zhu J., Fan D. A review on the study on MQTT security challenge. Proceedings of the 2020 IEEE International Conference on Smart Cloud (SmartCloud).

[13] Ashraf S., Shawon M.H., Khalid H.M., Muyeen S.M. (2021). Denial-of-Service Attack on IEC 61850-Based Substation Automation System: A Crucial Cyber Threat towards Smart Substation Pathways. Sensors.

[14] Khalid H.M., Muyeen S.M., Peng J.C.H. (2020). Cyber-Attacks in a Looped Energy-Water Nexus: An Inoculated Sub-Observer-Based Approach. IEEE Syst. J..

[15] Musleh A.S., Khalid H.M., Muyeen S.M., Al-Durra A. (2019). A Prediction Algorithm to Enhance Grid Resilience Toward Cyber Attacks in WAMCS Applications. IEEE Syst. J..

[16] Khalid H.M., Peng J.C.H. (2016). A Bayesian algorithm to enhance the resilience of WAMS applications against cyber attacks. IEEE Trans. Smart Grid.

[17] Esfahani A., Mantas G., Ribeiro J., Bastos J., Mumtaz S., Violas M.A., Duarte A.M.D.O., Rodriguez J. (2019). An efficient web authentication mechanism preventing man-in-the-middle attacks in industry 4.0 supply chain. IEEE Access.

[18] Abomhara M., Køien G.M. (2015). Cyber Security and the Internet of Things: Vulnerabilities, Threats, Intruders and Attacks. J. Cyber Secur. Mobil..

[19] Ghazi D.S., Hamid H.S., Zaiter M.J., Ghazi Behadili A.S. (2024). Performance and efficacy of Snort versus Suricata in intrusion detection: A benchmark analysis. AIP Conf. Proc..

[20] Yang Z., Liu X., Li T., Wu D., Wang J., Zhao Y., Han H. (2022). A systematic literature review of methods and datasets for anomaly-based network intrusion detection. Comput. Secur..

[21] Inayat U., Zia M.F., Mahmood S., Khalid H.M., Benbouzid M. (2022). Learning-Based Methods for Cyber Attacks Detection in IoT Systems: A Survey on Methods, Analysis, and Future Prospects. Electronics.

[22] Khraisat A., Alazab A. (2021). A critical review of intrusion detection systems in the internet of things: Techniques, deployment strategy, validation strategy, attacks, public datasets and challenges. Cybersecurity.

[23] Meena G., Choudhary R.R. A review paper on IDS classification using KDD 99 and NSL KDD dataset in WEKA. Proceedings of the 2017 International Conference on Computer, Communications and Electronics (COMPTELIX).

[24] Liu J., Kantarci B., Adams C. Machine Learning-driven intrusion detection for Contiki-NG-Based IoT networks exposed to NSL-KDD dataset. Proceedings of the 2nd ACM Workshop on Wireless Security and Machine Learning.

[25] Fatani A., Dahou A., Al-Qaness M.A., Lu S., Elaziz M.A. (2022). Advanced feature extraction and selection approach using deep learning and aquila optimizer for iot intrusion detection system. Sensors.

[26] Kolias C., Kambourakis G., Stavrou A., Gritzalis S. (2016). Intrusion detection in 802.11 networks: Empirical evaluation of threats and a public dataset. IEEE Commun. Surv. Tutor..

[27] Rahman M.A., Asyhari A.T., Wen O.W., Ajra H., Ahmed Y., Anwar F. (2021). Effective combining of feature selection techniques for machine learning-enabled IoT intrusion detection. Multimed. Tools Appl..

[28] Abdalgawad N., Sajun A., Kaddoura Y., Zualkernan I.A., Aloul F. (2022). Generative Deep Learning to detect cyberattacks for the IoT-23 dataset. IEEE Access.

[29] Hindy H., Bayne E., Bures M., Atkinson R., Tachtatzis C., Bellekens X., Ghita B., Shiaeles S. (2021). Machine Learning Based IoT Intrusion Detection System: An MQTT Case Study (MQTT-IoT-IDS2020 Dataset). Lecture Notes in Networks and Systems, Proceedings of the Selected Papers from the 12th International Networking Conference, Virtual, 21 September 2020.

[30] Ullah I., Mahmoud Q.H. (2021). A Framework for Anomaly Detection in IoT Networks Using Conditional Generative Adversarial Networks. IEEE Access.

[31] Koroniotis N., Moustafa N., Sitnikova E., Turnbull B. (2019). Towards the development of realistic botnet dataset in the Internet of Things for network forensic analytics: Bot-IoT dataset. Future Gener. Comput. Syst..

[32] Peterson J.M., Leevy J.L., Khoshgoftaar T.M. A review and analysis of the bot-iot dataset. Proceedings of the 2021 IEEE International Conference on Service-Oriented System Engineering (SOSE).

[33] Raikar M.M., Meena S. Vulnerability assessment of MQTT protocol in Internet of Things (IoT). Proceedings of the 2021 2nd International Conference on Secure Cyber Computing and Communications (ICSCCC).

[34] Alaiz-Moreton H., Aveleira-Mata J., Ondicol-Garcia J., Muñoz-Castañeda A.L., García I., Benavides C. (2019). Multiclass classification procedure for detecting attacks on MQTT-IoT protocol. Complexity.

[35] García-Ordás M.T., Aveleira-Mata J., Casteleiro-Roca J.L., Calvo-Rolle J.L., Benavides-Cuellar C., Alaiz-Moretón H., Analide C., Novais P., Camacho D., Yin H. (2020). Autoencoder Latent Space Influence on IoT MQTT Attack Classification. Proceedings of the Intelligent Data Engineering and Automated Learning–IDEAL.

[36] Aveleira-Mata J., Muñoz-Castañeda Á.L., García-Ordás M.T., Benavides-Cuellar C., Benítez-Andrades J.A., Alaiz-Moretón H. (2021). IDS prototype for intrusion detection with machine learning models in IoT systems of the Industry 4.0. Dyna.

[37] Zhang H. (2025). Development of an intelligent intrusion detection system for IoT networks using deep learning. Discov. Internet Things.

[38] Kandhro I.A., Alanazi S.M., Ali F., Kehar A., Fatima K., Uddin M., Karuppayah S. (2023). Detection of real-time malicious intrusions and attacks in IoT empowered cybersecurity infrastructures. IEEE Access.

[39] Lakshminarayana S., Praseed A., Thilagam P.S. (2024). Securing the IoT application layer from an MQTT protocol perspective: Challenges and research prospects. IEEE Commun. Surv. Tutor..

[40] ul Arfeen Laghari S., Li W., Manickam S., Nanda P., Al-Ani A., Karuppayah S. (2024). Securing MQTT Ecosystem: Exploring Vulnerabilities, Mitigations, and Future Trajectories. IEEE Access.

[41] Vaccari I., Aiello M., Cambiaso E. (2020). SlowITe, a Novel Denial of Service Attack Affecting MQTT. Sensors.

[42] Asgar S.A.G., Reddy N. (2025). Analysis of Misconfigured IoT MQTT Deployments and a Lightweight Exposure Detection System.

[43] Kant D., Johannsen A., Creutzburg R. (2021). Analysis of IoT Security Risks based on the exposure of the MQTT Protocol. Electron. Imaging.

[44] Aveleira-Mata J., Michelena Á., García-Rodríguez I., Calvo-Rolle J.L., Benavides C., Jove E. (2025). CoAP_UAD: CoAP under attack dataset - A comprehensive dataset for CoAP-based IoT security research. Data Brief.

[45] Chawla N.V., Bowyer K.W., Hall L.O., Kegelmeyer W.P. (2002). SMOTE: Synthetic Minority Over-sampling Technique. J. Artif. Intell. Res..

[46] Teixeira R., Almeida L., Rodrigues P., Antunes M., Gomes D., Aguiar R.L. (2025). Beyond performance comparing the costs of applying Deep and Shallow Learning. Comput. Commun..

[47] Xu Y., Zhou Y., Sekula P., Ding L. (2021). Machine learning in construction: From shallow to deep learning. Dev. Built Environ..

[48] Alexandre-Cortizo E., Rosa-Zurera M., Lopez-Ferreras F. Application of Fisher Linear Discriminant Analysis to Speech/Music Classification. Proceedings of the EUROCON 2005-The International Conference on “Computer as a Tool”.

[49] Ghojogh B., Crowley M. (2019). Linear and quadratic discriminant analysis: Tutorial. arXiv.

[50] Hastie T., Tibshirani R., Friedman J. (2009). The Elements of Statistical Learning.

[51] Friedman J.H. (1989). Regularized discriminant analysis. J. Am. Stat. Assoc..

[52] Rish I. An empirical study of the naive Bayes classifier. Proceedings of the IJCAI 2001 Workshop on Empirical Methods in Artificial Intelligence.

[53] Reddy E.M.K., Gurrala A., Hasitha V.B., Kumar K.V.R. (2022). Introduction to Naive Bayes and a review on its subtypes with applications. Bayesian Reasoning and Gaussian Processes for Machine Learning Applications.

[54] Kamel H., Abdulah D., Al-Tuwaijari J.M. Cancer classification using gaussian naive bayes algorithm. Proceedings of the 2019 International Engineering Conference (IEC).

[55] McCallum A., Nigam K. A comparison of event models for naive bayes text classification. Proceedings of the AAAI Conference on Artificial Intelligence.

[56] Cover T., Hart P. (1967). Nearest neighbor pattern classification. IEEE Trans. Inf. Theory.

[57] Kotsiantis S.B. (2013). Decision trees: A recent overview. Artif. Intell. Rev..

[58] Salzberg S.L. (1994). C4.5: Programs for machine learning by j. ross quinlan. morgan kaufmann publishers, inc., 1993. Mach. Learn..

[59] Mienye I.D., Jere N. (2024). A survey of decision trees: Concepts, algorithms, and applications. IEEE Access.

[60] Cutler A., Cutler D.R., Stevens J.R. (2012). Random forests. Ensemble Machine Learning.

[61] Ogunleye A., Wang Q.G. (2020). XGBoost Model for Chronic Kidney Disease Diagnosis. IEEE/ACM TRansactions Comput. Biol. Bioinform..

[62] Friedman J.H. (2001). Greedy function approximation: A gradient boosting machine. Ann. Stat..

[63] Baum E.B. (1988). On the capabilities of multilayer perceptrons. J. Complex..

[64] Murtagh F. (1991). Multilayer perceptrons for classification and regression. Neurocomputing.

